# Cerebral proliferative angiopathy in pediatric patients: case-based review with an illustrative case

**DOI:** 10.1007/s00381-026-07129-8

**Published:** 2026-02-06

**Authors:** Anzhela D. Moskalik, Jonathan Mo, Monifa Sawyerr, Marike Zwienenberg, Branden Cord, Julia D. Sharma

**Affiliations:** 1 Department of Neurological Surgery, University of California Davis Medical Center, 4860 Y Street, Sacramento, CA 95817 USA; 2 University of California Davis Medical Center, University of California School of Medicine, Sacramento, CA USA

**Keywords:** Cerebral proliferative angiopathy, Indirect revascularization, Pial synangiosis, Vascular steal syndrome

## Abstract

**Purpose:**

To synthesize pediatric presentations, imaging, and management of cerebral proliferative angiopathy (CPA) and to illustrate surgical decision-making with a representative case.

**Methods:**

We conducted a systematic search of CINAHL, Cochrane Library, Embase, Ovid MEDLINE, Scopus, and Web of Science from inception to February 2024 per PRISMA guidance, extracting demographics, presentation, lesion distribution, treatment, and outcomes from pediatric cases. We also detail a 2-year-old with hemispheric CPA treated by pial synangiosis plus burr-hole dural inversion.

**Results:**

Twenty-one studies comprising 29 pediatric CPA cases were included. Common symptoms were focal deficits (*n* = 17), headache (*n* = 15), and seizures (*n* = 6). Nidi most frequently involved frontal, temporal, and parietal lobes, with more bilateral and infratentorial involvement than mixed-age cohorts. Fifteen patients received conservative therapy; nine underwent surgery—most commonly indirect revascularization. Our case showed angiographic collateralization over the right motor cortex and absence of new infarction at 2-year follow-up, with functional gains and no recurrent ischemia.

**Conclusion:**

Pediatric CPA often manifests with ischemia and diffuse, eloquent-intermixed vasculature. In symptomatic children with hypoperfusion, indirect revascularization is a reasonable strategy to enhance perfusion and reduce recurrent ischemic events. Aggregated evidence and our illustrative case suggest robust and durable outcomes following pial-based indirect bypass, supporting early multidisciplinary evaluation and individualized surgical consideration.

## Introduction

Cerebral proliferative angiopathy (CPA) is a rare cerebral vascular disorder characterized by abnormal blood vessel proliferation interspersed among normal brain parenchyma [14]. CPA typically presents with seizures, headaches, and focal neurologic deficits that may occur due to disorganized angiogenesis secondary to hypoperfusion and ischemia [3, 20]. CPA is defined as an atypical entity that differs from classic brain arteriovenous malformations (AVMs) based on epidemiology, clinical presentation, angiographic and histopathologic features, and management [23]. With traditional AVMs, common treatments include surgical resection, embolization, and radiosurgery; however, in CPA, aggressive interventions may put patients at increased risk for neurological deficits due to abnormal vasculature being intermingled within functional brain tissue [23].

Since Lasjaunias et al. first defined CPA in 2008, several case reports have highlighted the occurrence of the disease primarily in young, adult patients [14]. Here, we present a case of CPA in a 2-year-old female patient who initially presented with acute neurologic symptoms of left-sided weakness, rightward gaze, head deviation, left-sided limping, and unsteady gait that led to a complete inability to walk. Initial imaging displayed multifocal acute infarcts and extensive vascular abnormalities in the right frontoparietal cortices. The patient was prescribed aspirin and levetiracetam due to increased seizure risk and later underwent indirect revascularization via pial synangiosis and multiple burr holes with dural inversion.

## Clinical presentation

Our patient was a 2-year-old previously healthy female who presented due to an acute onset of left-sided weakness, rightward gaze, and head deviation while playing hide and seek. Her parents noted that she appeared to start limping on the left side as well as stumbling and appeared imbalanced. She shortly progressed to a complete inability to walk. On presentation to the emergency department, her parents also noted that she appeared less active and talkative.

## Diagnosis

**Fig. 1:**
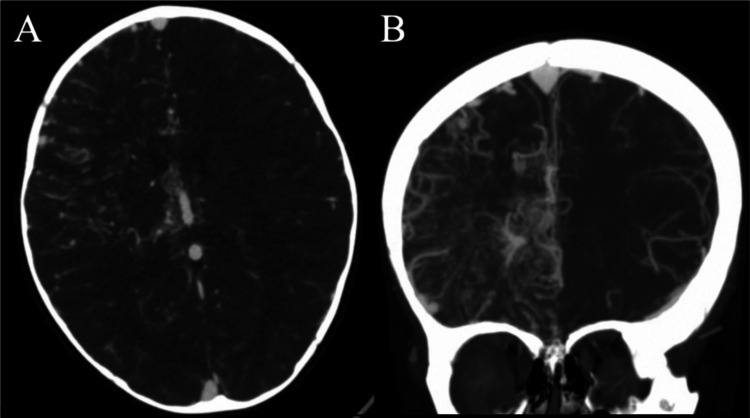
Initial computed tomography angiogram. **A** Axial and **B** coronal sequences showing increased vascularity in the right hemisphere concerning for a vascular malformation

**Fig. 2:**
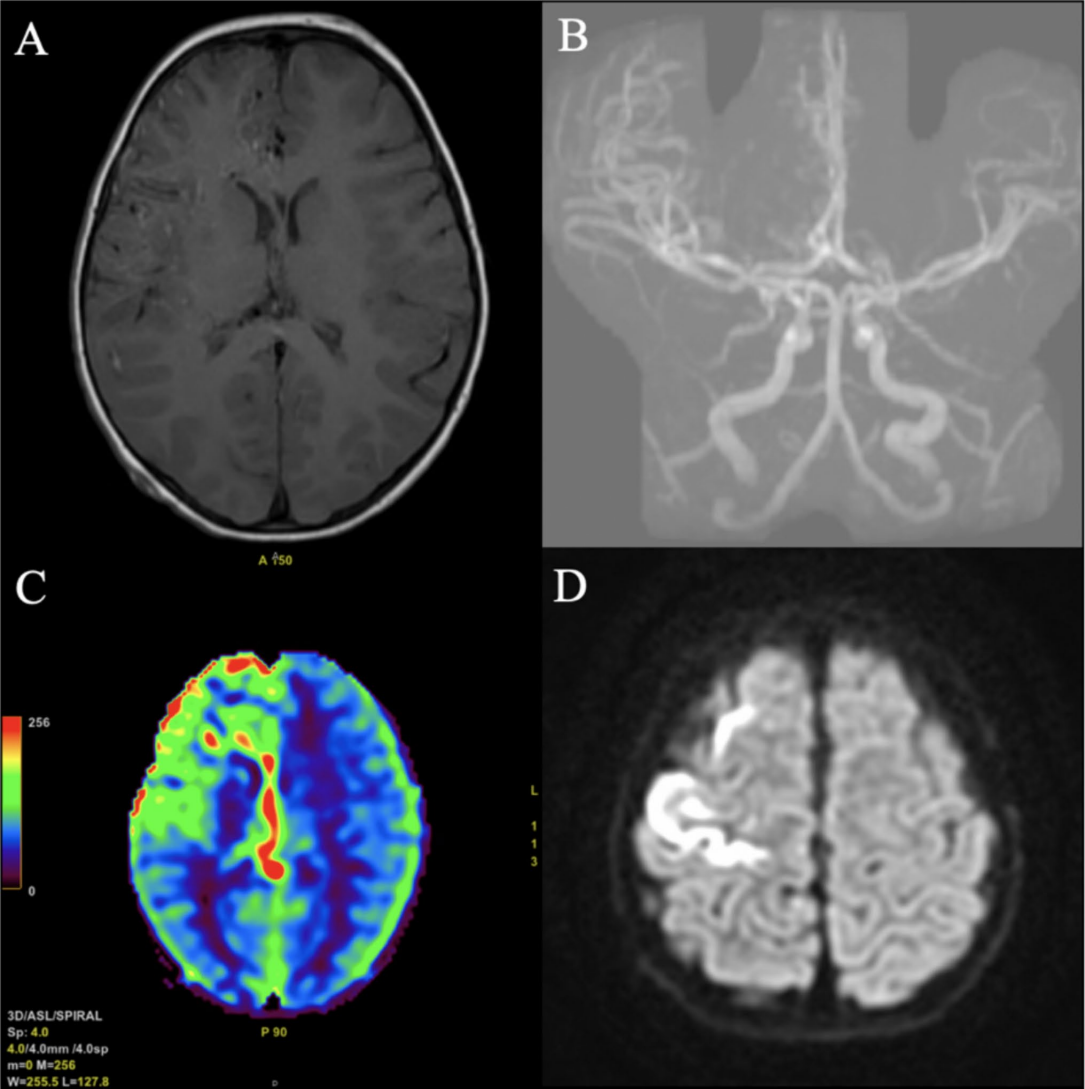
Magnetic resonance (MR) imaging including **A** axial gadolinium-enhanced sequence, **B** coronal MR angiogram with increased vascularity in the right hemisphere but no clear evidence of a vascular nidus/malformation. **C** Axial cerebral blood flow showing increased cerebral blood flow in the right frontal lobe. **D** Axial diffusion-weighted imaging with evidence of acute ischemia in the right frontal lobe and the pericentral cortex

**Fig. 3:**
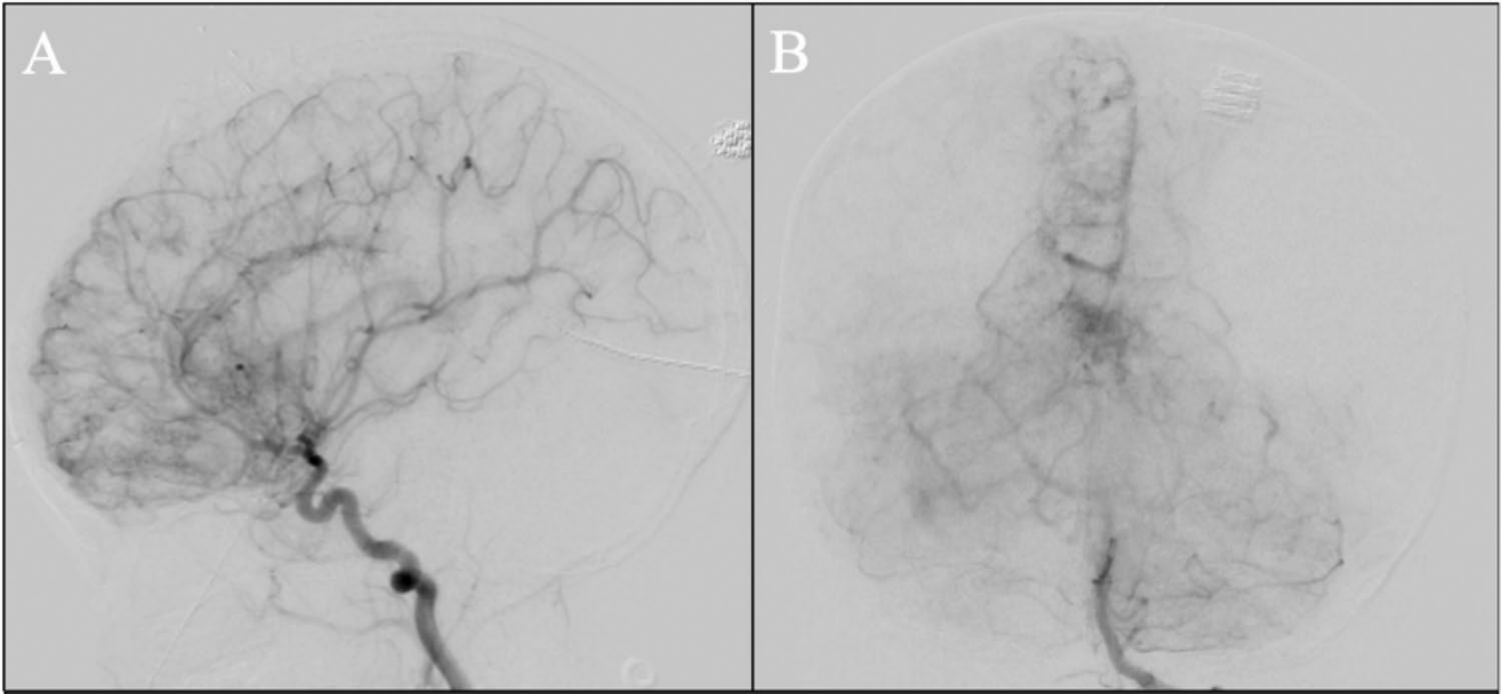
**A** Lateral right internal carotid artery and **B** anterior-posterior posterior circulation angiogram projections showing extensive proliferation of small vessels of the right frontal and parietal lobes with early venous drainage not in proportion to the degree of abnormal vasculature as well as increased small vessel vascularity noted in the thalamic vessels bilaterally. Findings most consistent with cerebral proliferative angiopathy

Initial imaging was significant for an unremarkable non-contrast computerized tomography (CT) of the head and a CT angiogram (CTA) of the head concerning for a right frontal vascular malformation (Fig. 1A, B). MRI of the brain was significant for multifocal acute infarcts within the right frontoparietal cortices with extensive increased vascular flow voids in the frontoparietal region with prominent enlarged veins draining to the superior sagittal sinus and right internal cerebral vein (Fig. 2). Upon evaluation, her neurological exam was significant for left hemiparesis (grade 3/5), left-sided neglect, and decreased tone in the left arm and leg. She was started on levetiracetam due to concern for seizure on presentation. A six-vessel digital subtraction cerebral angiogram was performed to better understand the vascular anatomy of the lesion. Selective injection of the right internal carotid artery (ICA) demonstrated innumerable vessels throughout the right frontal and parietal lobes that were associated with early venous drainage in a hypertrophied anterior cortical vein (Fig. 3A). There is an area of poor perfusion in the superior parietal area. Selective injection of the left ICA demonstrated normal filling of the left hemisphere but there was filling across the anterior communicating artery (ACOM) with evidence of a hypoplastic right anterior cerebral artery (ACA). Innumerable small vessels of the right ACA territory were noted and associated with some early venous drainage into the internal cerebral veins. Posterior circulation injection was significant for increased small vessel vascularity of the anterior border of the visualized territory. Additional increased small vessel vascularity was noted in the thalamic vessels bilaterally (Fig. 3B). Collectively, these findings are consistent with prior descriptions of cerebral proliferative angiopathy where hypoplastic arteries with early venous drainage are associated with hypoperfusion, which leads to angiogenesis presenting as numerous small vessels in the area of hypoperfusion [14].

## Management

The patient was discharged home shortly after on aspirin and levetiracetam. After a discussion with the parents, the patient underwent a delayed (at 3 months after presentation) indirect vascular bypass via pial synangiosis in addition to multiple burr holes with dural inversion to allow for hemispheric coverage.

A Doppler ultrasound was used to trace the course of the parietal and frontal branches of the superficial temporal artery (STA). The parietal branch was used for revascularization. A T-shaped incision was designed to obtain hemispheric exposure as well as to dissect and isolate the STA. Once 6 to 7 cm of the STA was isolated, we proceeded with a temporoparietal craniotomy. The dura was opened in a cruciate fashion. The arachnoid was opened, and the STA was secured to the pia with 10-0 nylon suture. An onlay duraplasty with synthetic dural substitute was performed, and the cranial flap was replaced and secured with silk suture. Next, we extended the incision in an anterior-posterior orientation and made multiple burr holes posterior to the craniotomy site. The dura was opened in a cruciate manner in each burr hole and reflected.

## Prognosis and outcomes

The patient initially recovered well postoperatively. The patient’s strength improved to 4/5 in the left arm and leg, and she regained her ability to walk, which we attribute to the natural course of recovery in a young child. Unfortunately, her clinical course was complicated, and she developed a small wound breakdown requiring revision as well as a pseudomeningocele requiring cerebral spinal fluid (CSF) diversion with a ventriculoperitoneal shunt. We believe that the patient developed abnormal CSF fluid dynamics secondary to the lack of water-tight dural closure in addition to the increased congestion of the dural venous system leading to increased intracranial pressure [15, 16].

**Fig. 4:**
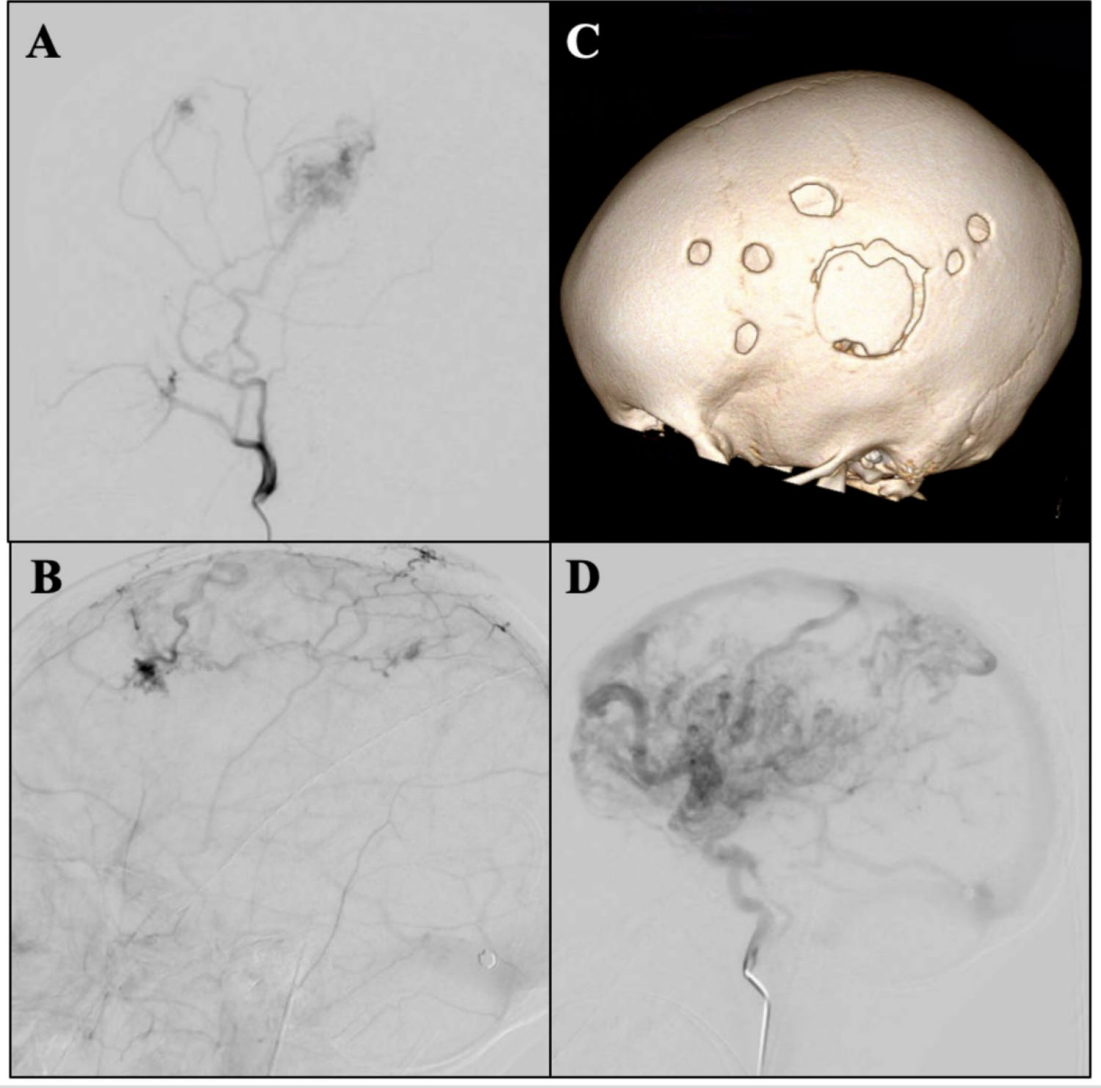
**A** Lateral right ICA and **B** lateral right ECA angiogram projections showing proliferation of small vessels through the burr holes as well as a successful EDAS over approximately the right motor cortex compared to the three-dimensional model (**C**) based on a post-operative CT scan. **D** Lateral right ICA injection showing innumerable vessels with early venous drainage into hypertrophied veins

The patient was doing well on her 2-year post-operative follow-up with no recurrence of symptomatic cerebral ischemia and no recurrent seizures. A surveillance diagnostic angiogram and an MRI were performed at that time. Selective injection of the right ECA showed a patent right EDAS that approximately overlies the motor cortex with good perfusion (Fig. 4A, C). There are areas of anastomosis with intracranial blood supply which correspond to additional burr holes (Fig. 4A, B, C). The right ICA injection was significant for innumerable vessels throughout the right frontal and parietal lobes with early venous drainage and vein hypertrophy (Fig. 4D). No new infarctions were noted on the MRI.

## Exemplary case description

CPA is a rare vascular lesion first described by Lasjaunias et al. in 2008 and differs from traditional AVMs in their natural history, clinical course, angioarchitecture, and radiographic features [14]. CPA is estimated to account for 2–4% of all cerebral AVMs and more commonly presents in young, adult females with a mean age of symptom onset of 22 years [2, 14]. Current literature on CPA mostly draws from case reports and series of adult patients with few descriptions of pediatric cases. Here we report a case of CPA at our institution and provide a systematic review of the literature with a focus on the pediatric population (Table 1).

**Table 1:** Reported cases of cerebral proliferative angiopathy within the pediatric population

Author and year	Age (years)/Sex	Presentation	Imaging obtained	Radiographic findings	Medical management	Surgical management	Time to operation	Outcome
Our patient	2/F	Left-sided weakness, rightward gaze, head deviation	CTA, MRI, Angiogram	Multifocal acute infarcts within right frontoparietal cortices, increased vascular flow voids in the frontoparietal region with enlarged veins draining to superior sagittal sinus and right ICA. Innumerable vessels throughout right frontal and parietal lobes with early venous drainage and a hypertrophied anterior cortical vein; additional filling across ACOM with a hypoplastic right ACA	Aspirin, Levetiracetam	Indirect Revascularization (pial synangiosis), Multiple burr holes with dural invasion	3 months	2-year follow-up was stable with no recurrence
Beniwal 2020 [1]	12/M	Drowsiness without focal neurological deficits	CT, MRI/MRA, Angiogram	Right cerebellar parenchymal hemorrhage with mass effect over 4th ventricle, mild cerebral edema, ill-defined cluster of vessels with intervening normal parenchyma. Ill-defined lesion in right cerebellar hemisphere fed by SCA, AICA, PICA	Symptomatic management (unspecified)	None	None	18-month follow-up was stable
Chen 2022 [4]	6/M	Repetitive episodes of sudden LOC, Right hemiplegia	MRI, MRI perfusion, Angiogram	Diffuse, dilated vessels throughout entire left cerebral hemisphere intermingled with normal parenchyma, clustered dilated vessels in left basal ganglia and thalamus. Hypoperfusion of left hemisphere except in basal ganglia. Numerous AV shunts composed of tiny feeding arteries and multiple deep draining veins	Unspecified	Indirect Revascularization (EDAS) + Dural infoldings, followed by SRS	SRS at 9 months and 2 years post-EDAS	Stable at 4 years post-op, CPA nidus shrinkage, good collateral formation
Choi 2021 [5]	11/M	Epilepsy with LOC and aphasia	MRI/MRA, Angiogram	Large nidus supplied by multiple arteries stemming from left MCA and lenticulostriate artery	Valproate, Topiramate	Surgical excision	3 months	3 years, no recurrence, shrinkage of nidus
Ellis 2011 [6]	2/F	Left-sided hemiplegia and left-sided facial droop	CT, MRI, Angiogram	Diffuse, extensive vascular lesion associated with extensive T2 signal change in right frontal, temporal, and parietal regions. Significant associated encephalomalacia and atrophy. 4.5 cm vascular malformation with complex arterial supply involving bilateral internal carotid arteries, ACA, MCA, PCA, right MMA, and left occipital artery	Unspecified, Underwent rehabilitation (3-month duration) for motor deficits	Indirect Revascularization: pial synangiosis, encephalomyosynangiosis, and dural inversion	> 2 years	1-year follow-up with improvement of weakness. Imaging with revascularization of right hemisphere
Fierstra 2011 [7]	11/F	Seizures		NA	Unspecified	Unspecified	Unspecified	NA
Fierstra 2011 [7]	11/M	TIA		NA	Unspecified	Unspecified	Unspecified	NA
Gatto 2018 [8]	15/F	5 years of headaches and right hemiparesis	MRI, Angiogram	Bulky, vascular malformation affecting entire left hemisphere fed by right ACA, MCA, PCA with superficial venous drainage, without flow or intranidal aneurysms	Symptomatic/conservative management (unspecified)	None	None	NA
Giragani 2018 [9]	12/M	Headache, vomiting, and neck pain	CT, MRI	Right cerebellar hemorrhage with intraventricular extension causing hydrocephalus. Diffuse, ill-defined vascular blush in posterior fossa with arterial feeders from bilateral PCA, SCA, PICA, AICA	Unspecified	Endovascular embolization with ETV	Unspecified	6-month follow-up was stable
Gold 2013 [10]	9/F	Headaches, right hemiparesis & homonymous hemianopia	CT, CTA	Left hemisphere vascular malformation, without evidence of dural AV fistula. Engorged sagittal and straight sinus with prominent cortical veins	Unspecified	None	None	Symptoms resolved, at 1 year: similar presentation with symptom resolution
Karian 2017 [11]	7/F	Headache, left hemiparesis	MRI, Angiogram	Large right parieto-occipital vascular malformation. Focal AV shunting	Aspirin	None	None	NA
Khan 2013 [12]	16/F	Headaches, Right-sided weakness	MRI, Angiogram	Diffuse network of densely enhancing vascular spaces over left parietal region intermingled with normal brain parenchyma. Widespread AVM supplied by ACA, MCA, lenticulostriate arteries, and diffuse network of vascular spaces with relatively low shunting	Unspecified	None	None	NA
Kimiwada 2013 [13]	13/M	Headaches and right upper limb numbness	MRI, perfusion, Angiogram, SPECT	Diffuse vascular lesion in sulci of left frontal and parietal lobes. Increased CBV/CBF within lesion. Hypervascular lesion with diffuse arterial supply involving left ACA, MCA, PCA, MMA	Carbamazepine	None	None	Symptoms improved, 1-year follow-up w/no deficits
Liu 2016 [18]	4/F	Severe headaches	MRI, PET, Angiogram	Hypervascular lesion with early venous filing involving almost the entire right hemisphere. Right ECA showed dural blood supply to the intracranial lesion. Severely impaired perfusion	Conservative management (Observation)	None	None	NA
Liu 2016 [18]	5/F	Headaches and left intermittent limb weakness	MRI, Angiogram, PET	Diffuse network of densely enhancing vascular spaces with intermingled normal brain parenchyma. Absence of dominant feeders. Decreased perfusion of nidus in the surrounding tissue	Conservative management (Observation)	None	None	Persistent left limb weakness at 12-month follow-up
Luo 2023 [19]	6/F	Two-year transient unsteadiness and slurred speech	MRI/MRA, Angiogram	Numerous anomalous hyperplastic capillaries in area supplied by the internal carotid arteries and PCA bilaterally, several branches of external carotid arteries and dural vessels supplying blood to intracranial area	Conservative management (sedation, fluid replacement, anticoagulation)	None	None	3-month follow-up with no symptom change
Ochoa 2022 [20]	5/X	Headaches, vomiting, drowsiness	CT, Angiogram	Intraparenchymal hemorrhage with in right temporoparietal region. Large malformed nidus in right temporal lobe with intermingled brain parenchyma, arterial feeders, draining veins of small size relative to large nidus	Conservative management following discovery of CPA	Two hematoma evacuations, anterior temporal lobectomy to remove the nidus	2 years	2 year follow up with 2 episodes of asymptomatic re-hemorrhage
Ochoa 2022 [20]	16/F	Sudden headache and drowsiness	CT	Right cerebellar hematoma with compromise of perimesencephalic cisterns	Unspecified	CPA excision with portion of right cerebellar hemisphere	2 months	3-year follow-up demonstrated no sequelae and complete recovery
Puerta 2017 [21]	8/M	Left hemiparesis, headaches, and transient ischemic attacks	MRI, CTA	Diffuse network of densely enhancing vascular spaces intermingled with normal brain parenchyma. Widespread angiopathy fed by multiple arteries and diffuse network of vascular spaces with slow shunting	Unspecified	Indirect revascularization: right-sided pial synangiosis and encephalomyosynangiosis	Around 1 year	1-year follow-up: no deficits, 2-year follow-up: permanent left hemiparesis with gait disturbance
Rebello 2021 [22]	12/M	Tonic-clonic seizure and sudden onset left hemiplegia	CT, Angiogram	Right frontal lobe hemorrhage, old infarct in right anterior frontal lobe. Arterial phase demonstrated abnormal nidus of vessels in right frontal and parietal regions fed by cortical branches of MCA with no draining vein appreciated	Conservative management with physiotherapy	None	None	2-month follow-up showed improved modified Rankin Scale score (of 1)
Sakata 2016 [23]	8/M	Transient ischemic attack leading to left extremity motor weakness	MRI, SPECT, Angiogram	Marked proliferation and dilatation of pial vessels in right hemisphere. Hemodynamic compromise of right hemisphere. Diffuse large vascular malformation with two structures: 1. AVF in right basal ganglia, 2. surrounding proliferated pial/medullary arteries in right hemisphere	Conservative management (unspecified) prior to surgery	Extracranial-intracranial revascularization (extended burr hole surgery), then embolization of right A1 pseudoaneurysm	7 years/Embolization procedure performed 3.5 months after revascularization	2-year follow-up with no deficits, MRA with revascularization from MMA, STA, and deep temporal artery
Singfer 2023 [25]	6/F	Right retro-orbital headache, diplopia, nausea	CT, MRI, Angiogram	Diffuse vascular network supplied by branches of right MCA/PCA, enlarged veins of superior sagittal sinus and internal cerebral vein. No dominant arterial feeders, no proximal arterial stenosis. Persistent opacification of malformation in late arterial phase and some early venous filling	Conservative management (unspecified)	None	None	6-year follow-up: intermittent symptoms. MRI w/slow progression of malformation
Srivastava 2021 [26]	14/F	Headache, seizures, acute left-sided weakness	MRI, CT	Flow voids in right frontoparietal area with patchy contrast enhancement and restricted diffusion. Severe stenosis of right MCA and diffuse proliferative angiopathic changes, no dominant feeders or early draining veins, presence of transdural feeders	Aspirin, Anti-epileptic	None	None	Lost to follow-up
Srivastava 2022 [27]	17/M	Seizures		NA	Aspirin, Anti-epileptic	None	None	1-month follow-up was stable
Srivastava 2022 [27]	17/F	Headache and papilledema		NA	Aspirin, Anti-epileptic	None	None	2-year follow-up was stable
Srivastava 2022 [27]	17/M	Seizure, hemiparesis,		Hemorrhage in left frontoparietal region	Aspirin, Anti-epileptic	None	None	Died within 1 year
Srivastava 2022 [27]	14/F	Headache, seizure, hemiparesis		NA	Aspirin, Anti-epileptic	None	None	1 month follow-up was stable
Vargas 2011 [28]	14/M	Diminished sensation and spasticity on the right, expressive aphasia	MRI, Angiogram	Extensive vascular malformation with presence of flow voids, intermingled brain associated with parenchymal atrophy. Increased right CBV/CBF, delayed MTT	Unspecified	Unspecified	Unspecified	NA
Vargas 2011 [28]	17/F	Headache and personality changes	MRI, Angiogram	Diffuse network of vessels intermingled with brain parenchyma, Abnormal flow voids in left temporal, parietal, and occipital lobes. Increased CBF/CBV, prolonged MTT, decreased TTP, little AV shunting present compared to size of malformation, no significantly large arterial feeders or flow-related aneurysms	Symptomatic management (unspecified)	None	None	NA

### Pathophysiology

While the underlying pathophysiology of CPA is not well understood, the commonly accepted hypothesis posits that CPA develops as a consequence of the vascular steal phenomenon: low-resistance arteriovenous shunting within the lesion decreases arterial blood pressure in perinidal brain tissue, leading to arterial autoregulatory mechanisms that attempt to maintain regional blood flow. With even greater decreases in perinidal perfusion, compensatory autoregulatory mechanisms are not enough to maintain regional flow, thus leading to hypoperfusion-induced angiogenesis [6, 13]. Imaging and laboratory studies support this hypothesis, further distinguishing the pathophysiological differences between CPA and AVMs. Fierstra et al. demonstrated severely impaired perilesional cerebrovascular reserve in patients with CPA compared to patients with AVMs and healthy individuals using blood oxygen level-dependent magnetic resonance imaging [7]. With perfusion-weighted MRI, Saliou et al. [24] found increased cerebral blood volume, mean transit time, and microvascular leakage in patients with CPA compared to those with AVMs, pointing to a pattern of increased angiogenesis [22]. Cerebrospinal fluid studies in an adult case of CPA demonstrated high levels of vascular endothelial growth factor, thrombospondin, and basic fibroblast growth factor [18]. Taken together, these findings suggest that CPA forms as a response to chronic parenchymal oligemia, resulting in uncontrolled vascular proliferation.

### Demographics

Lasjaunias et al. first described demographic characteristics of CPA with a retrospective database review of 49 mixed adult and pediatric patients [14]. Our literature review, focusing exclusively on pediatric CPA cases, demonstrates an average age at diagnosis of 10.6 years with a median age at diagnosis of 11 years. Sixteen cases involved female patients, 12 involved male patients, and one case did not report gender. After initial medical or surgical intervention, the average total follow-up time, defined as the longest follow-up time reported in each case, was 19.8 months (as reported by 19 cases, with a range of 1 to 72 months). Compared with other pediatric reports, our 2-year-old patient is the youngest reported case of CPA along with the 2-year-old patient described by Ellis et al. [6].

### Presentation

CPA can present with a wide range of symptoms in children. In our review, two patients presented with loss of consciousness, four with altered mental status, four with transient neurological deficits, six with seizures, 15 with headaches (ranging from mild to disabling), and 17 with focal neurological deficits (most commonly hemiparesis, hemiplegia, or aphasia). As reported in the literature, CPA is more likely to present due to its ischemic manifestations (17% of patients versus 2% in patients with AVMs), but hemorrhagic manifestations are also not uncommon [1]. The rates of hemorrhage are overall lower in CPA compared with traditional AVMs (18% versus 50%), but the chances of rebleed after an initial hemorrhage are potentially as high as 67% [14, 17, 29]. Five pediatric patients presented with hemorrhage (17%), which is similar to previously reported rates of hemorrhagic CPA in mixed-age cohorts [14, 29]. Seizures are also a common presenting symptom in CPA, occurring in 45% of patients in the Lasjaunias et al. series compared to 16% in the overall AVM population. In our review, 29% of pediatric patients with CPA experienced seizures, and the actual rate may be even higher as there were reports of transient neurologic deficits in five other patients with no documentation as to whether an EEG was performed.

### Imaging characteristics and histopathology

CPA is characterized by distinct features on cross-sectional and angiographic imaging. These often include a large, diffuse nidus which is often lobar or hemispheric, an absence of dominant arterial feeders to the nidus or flow-related aneurysms, non-focal angiogenetic activity, such as the presence of extensive transdural supply, small draining veins relative to the arteriovenous shunting zone, capillary angioectasia, and proximal artery stenosis [14]. On histopathology, CPA lesions may demonstrate altered internal elastic lamination of arterial and venous structures, as well as collagenous thickening of the venous vasculature [27]. The presence of neural tissue intermingled between vascular channels is a distinctive feature of this disease and is consistently seen with both imaging and histopathology [14].

CPA lesions in our review were located in the left hemisphere in 11 patients (38%), the right hemisphere in 12 patients (41%), bilaterally in three patients (10%), and infratentorially in three patients (10%). Lobar involvement is as follows: frontal lobe, 11 (38%); temporal lobe, 3 (10%); parietal lobe, 11 (10%); occipital lobe, 2 (7%). CPA lesions affected the basal ganglia and thalamus in six patients (21%). Compared with the cohort described by Lasjaunias et al., we found greater bilateral hemispheric and infratentorial nidal involvement, and lesser involvement of the temporal lobe, parietal lobe, occipital lobe, and basal ganglia/thalamus [14].

### Treatment

Pediatric patients with CPA received a wide variety of treatments: 15 cases were treated conservatively, nine received surgical intervention, and five did not report a treatment plan. For the 15 patients receiving medical management, five were prescribed anti-epileptic medications, six were prescribed aspirin, and eight received unspecified symptomatic treatment. Four patients underwent indirect revascularization procedures, with three receiving pial synangiosis, two receiving encephalomyosynangiosis (EMS), and one receiving encephaloduroarteriosynangiosis (EDAS). Additionally, three patients had surgical excision of the CPA nidus or lobectomy, one received direct revascularization (direct extracranial-intracranial bypass), two underwent embolization procedures, and one received stereotactic radiosurgery. For patients undergoing surgery, the average time to operation from initial presentation was 21 months (with a median of 12 months and a range of 1 month to 7 years). Three patients who underwent surgical treatment had initial medical treatment: our patient received both anti-platelet and anti-seizure medication for 3 months, the patient described by Choi et al. received anti-seizure medications for 3 months [5], and the patient described by Ellis et al. underwent 3 months of physical rehabilitation [6]. Similar to the reasons stated by Choi et al. and Ellis et al., we opted for surgical intervention due to the persistence of neurological symptoms and widespread hypoperfusion secondary to the CPA lesion. The goal of surgery was to provide additional blood flow to the affected area to prevent further episodes of cerebral ischemia.

Ten of the 15 patients receiving medical management reported outcomes at follow-up. Eight patients were stable (mean follow-up of 9 months, median of 7.5 months), one had slow progression of their CPA malformation over a 6-year period, and one passed away within a year of initial presentation. For surgically managed patients, eight patients were stable at their last follow-up (mean follow-up of 24 months, median of 24 months) and one patient progressed to permanent left hemiparesis at their 2-year follow-up. For the patient with permanent hemiparesis, they had improvement in their symptoms and improved quality of life at the first year of follow-up. The authors suggest that hemiparesis may have occurred due to the eventual ischemia of the interspersed brain tissue within the CPA nidus; however, no further imaging or clinical specifications were reported [21].

We believe that indirect revascularization techniques should be considered for symptomatic pediatric CPA patients. While both direct and indirect revascularization may improve blood flow to hypoperfused brain regions, direct revascularization techniques are often impractical for pediatric populations [6]. After pial synangiosis with multiple burr holes and dural inversion, our patient demonstrated markedly improved motor function and was stable at her 1- year follow-up without any new ischemic events. Other pediatric patients undergoing indirect revascularization procedures demonstrated favorable findings as well. Ellis et al. reported that their patient’s 8-month post-operative angiogram demonstrated a stable vascular malformation with robust revascularization of the previously hypoperfused frontal and parietal lobes [6]. Chen et al. reported good collateral vasculature formation and improved left cerebral hemisphere perfusion that was stable 43 months after revascularization [4]. Similar to our patient, each of these patients showed improved weakness and no further transient neurological deficits by their last reported follow-up. It is unclear if the improvement in neurological function is related to the revascularization surgery versus a normal clinical course in pediatric patients; we do believe that the lack of new ischemic events points to improvement in blood flow secondary to the surgical intervention.

## Conclusions

CPA can present with varied symptomology and severity; however, children presenting with debilitating ischemic symptoms should be evaluated for surgical intervention by indirect revascularization. Indirect revascularization techniques can help facilitate neovascularization of hypoperfused brain regions affected by CPA-related vascular steal and have shown robust and durable outcomes in our patient and in the reviewed literature.

## Data Availability

All data are included within the article and Table 1.

## Abbreviations

ACA: Anterior cerebral artery
ACOM: Anterior communicating artery
AICA: Anterior inferior cerebellar artery
AV: Arteriovenous
CBF: Cerebral blood flow
CBV: Cerebral blood volume
CPA: Cerebral proliferative angiopathy
CT: Computed tomography
CTA: Computed tomography angiography
DSA: Digital subtraction angiography
ECA: External carotid artery
EDAS: Encephaloduroarteriosynangiosis
ETV: Endoscopic third ventriculostomy
ICA: Internal carotid artery
LOC: Loss of consciousness
MCA: Middle cerebral artery
MMA: Middle meningeal artery
MRI: Magnetic resonance imaging
MRA: Magnetic resonance angiography
MTT: Mean transit time
PCA: Posterior cerebral artery
PET: Positron emission tomography
PICA: Posterior inferior cerebellar artery
SCA: Superior cerebellar artery
SPECT: Single-photon emission computed tomography
SRS: Stereotactic radiosurgery
STA: Superficial temporal artery
TTP: Time to peak
